# Corneal Endothelial Changes in Primary Glaucoma: A Qualitative and Quantitative Evaluation by Disease Subtype and Antiglaucoma Medication Burden

**DOI:** 10.7759/cureus.95432

**Published:** 2025-10-26

**Authors:** Pravin Dhoriyanee, Seema Meena, Kavita R Bhatnagar, Nikhil Agrawal, Manjari Tandon, Suwarna Suman, Kirti Jaisingh, Jyoti Shakrawal

**Affiliations:** 1 Ophthalmology, All India Institute of Medical Sciences, Jodhpur, Jodhpur, IND

**Keywords:** antiglaucoma medications, corneal endothelium, endothelial cell density, glaucoma, primary angle closure glaucoma, primary open angle glaucoma

## Abstract

Introduction

Corneal endothelial cells (CECs) are critical for maintaining corneal clarity but have limited regenerative potential. Glaucoma and its treatment may compromise endothelial integrity. This study evaluated corneal endothelial changes in primary glaucoma, stratified by disease subtype and antiglaucoma medication (AGM) burden.

Methods

This cross-sectional study included 60 eyes of 34 patients with primary glaucoma and 60 eyes of 30 age- and sex-matched controls. Corneal endothelial parameters were assessed with a Tomey EM-4000 specular microscope. Patients were categorized as primary open-angle glaucoma (POAG) or primary angle-closure glaucoma (PACG) and further subgrouped by the number of topical AGMs.

Results

Glaucoma patients had lower endothelial cell density (ECD) (2325.86 ± 378.6 vs 2527.08 ± 197.05 cells/mm²; *P* < 0.001) and larger average cell size (*P* < 0.001) compared to controls. Both POAG (2405.74 ± 335.74 cells/mm²) and PACG (2177.52 ± 416.12 cells/mm²) groups had significantly reduced ECD compared to controls, with PACG showing greater loss than POAG (*P* = 0.02). ECD decreased with increasing AGM burden: 2,507.5 ± 189.89 cells/mm² (one AGM), 2460.85 ± 267.02 cells/mm² (two AGMs), and 2248 ± 383.44 cells/mm² (three AGMs) (*P* = 0.038).

Conclusion

Primary glaucoma is associated with significant corneal endothelial compromise, with PACG showing greater vulnerability than POAG. Increasing AGM burden correlated with further endothelial loss, suggesting possible AGM-related endothelial toxicity. Larger longitudinal studies are needed to clarify these effects.

## Introduction

The corneal endothelium consists of a monolayer of hexagonal cells that plays a crucial function in controlling stromal hydration and preserving corneal clarity via its pump mechanism [1-3]. Corneal endothelial cells (CECs) possess a restricted ability for in vivo regeneration, preventing them from replacing dead or damaged cells [4]. When endothelial cell density (ECD) declines below a critical threshold or under physiological stress, the cornea's functional reserve may be insufficient to maintain optimal hydration and clarity. There is growing evidence that glaucoma constitutes one of the several conditions that harm CECs [5].

Glaucoma is a potentially sight-threatening condition linked with optic neuropathy and retinopathy, as well as its impact on CECs [6]. The loss of endothelial cells in glaucoma is attributed to the compressive effect of elevated intraocular pressure (IOP), the toxicity of pharmacological treatments, and the consequences of surgical and laser interventions aimed at managing IOP [7-9]. Furthermore, angle closure may inflict damage on the endothelium due to iridocorneal contact or obstruction of aqueous flow, resulting in hypoxia and diminished nutritional support for the endothelium [9]. Glaucoma and cataracts are increasingly widespread and frequently coexist in the elderly population. Assessing CECs is essential in glaucoma patients to evaluate their capacity to endure the stress associated with cataract surgery, as CEC loss is an unavoidable outcome.

This study aimed to assess qualitative and quantitative changes in corneal endothelium in primary glaucoma, correlating these changes with IOP by comparing them to age- and sex-matched controls. Additionally, it aimed to assess the effect of antiglaucoma medications (AGMs) on the corneal endothelium in patients with primary glaucoma.

## Materials and methods

This prospective, cross-sectional study was conducted at the Department of Ophthalmology, All India Institute of Medical Sciences (AIIMS), Jodhpur, Jodhpur, Rajasthan, India, between July 2022 and December 2023, following approval from the Institutional Ethics Committee (Approval No. AIIMS/IEC/2022/4145). The study adhered to the tenets of the Declaration of Helsinki. Written informed consent was obtained from all participants prior to enrolment. A total of 120 eyes were evaluated, including 60 eyes from 34 patients diagnosed with primary glaucoma and 60 eyes from 30 age- and sex-matched healthy controls. Glaucoma cases were further classified into primary open-angle glaucoma (POAG) and primary angle-closure glaucoma (PACG) based on gonioscopic findings and optic nerve assessment.

Eligible participants were aged 40 years or older. Patients with secondary glaucoma, prior intraocular surgery, corneal pathology, ocular inflammation, or poor specular image quality were excluded. All subjects underwent a comprehensive ophthalmic examination, including best-corrected visual acuity (BCVA) using Snellen’s chart, IOP measurement via Goldmann applanation tonometry, slit-lamp biomicroscopy, gonioscopy using a Goldmann two-mirror lens, and dilated fundus examination with a 90D lens and indirect ophthalmoscopy. Visual field testing was performed using the Humphrey Field Analyzer (30-2 SITA Standard protocol). Glaucoma severity was graded as early, moderate, or severe according to the Hodapp-Parrish-Anderson criteria [10].

Corneal endothelial morphology was evaluated using the Tomey EM-4000 non-contact specular microscope. Parameters recorded included ECD (cells/mm²), average cell area ([AVG] µm²), coefficient of variation (CV, %), percentage of hexagonal cells (HEX %), and central corneal thickness (CCT, µm). Automated analysis was used for cell quantification, with manual correction applied when cell borders were indistinct. Continuous variables were expressed as mean ± standard deviation (SD). Intergroup comparisons were performed using independent sample t-tests and one-way ANOVA. All data were analyzed using IBM SPSS Statistics version 26 (IBM Corp., Armonk, NY, USA).

## Results

Demographic and clinical characteristics

A total of 120 eyes were evaluated, comprising 60 eyes of 34 patients with primary glaucoma (cases) and 60 eyes of 30 age- and sex-matched healthy individuals (controls). The mean age of the cases was 57.32 ± 10.72 years and that of the controls was 56.5 ± 11.47 years (P = 0.76). Among the cases, 24 (70.5%) were males and 10 (29.5%) were females, while in the controls, 22 (73.33%) were males and 8 (26.66%) were females.

Glaucoma subtypes and intraocular pressure

Among glaucoma cases, 39 (65%) had POAG and 21 (35%) had PACG. The mean IOP was significantly higher in cases (20.56 ± 7.72 mmHg) compared to controls (14.28 ± 2.31 mmHg; P < 0.001). PACG eyes exhibited significantly higher IOP than POAG eyes (23.52 ± 10.17 mmHg vs 18.97 ± 5.54 mmHg; P = 0.028).

Specular microscopy parameters: cases versus controls

The endothelial parameters assessed via specular microscopy included ECD, AVG, standard deviation of cell area (SD), CV, maximum and minimum cell area (MAX and MIN), hexagonality (HEX), and CCT. Mean ECD was significantly reduced in glaucoma cases (2325.86 ± 378.60 cells/mm²) compared to controls (2527.08 ± 197.05 cells/mm²; P < 0.001). The AVG was significantly higher in cases (443.48 ± 98.61 μm²) than in controls (397.41 ± 31.39 μm²; P < 0.001), and the SD was also elevated (174.00 ± 57.88 μm² vs 145.88 ± 20.14 μm²; P < 0.001), indicating increased polymegathism. CV was significantly greater in cases (38.93 ± 5.12%) compared to controls (36.93 ± 3.89%; P = 0.03). MAX was higher in cases (1123.40 ± 312.65 μm²) than in controls (958.40 ± 300.54 μm²; P = 0.02), and MIN was also elevated (109.86 ± 38.42 μm² vs 98.18 ± 15.36 μm²; P = 0.04). HEX was slightly reduced in cases (44.40 ± 6.41%) compared to controls (46.48 ± 5.54%; P = 0.05). CCT was comparable between groups (493.21 ± 36.61 μm in cases vs 503.05 ± 29.35 μm in controls; P = 0.10) (Table 1).

**Table 1:** Comparison of IOP and specular microscopic parameters between cases and controls AVG, average cell area; CCT, central corneal thickness; CV, coefficient of variation; ECD, endothelial cell density; HEX, percentage of hexagonal cells; IOP, intraocular pressure; MAX, maximum cell area; MIN, minimum cell area; SD, standard deviation *Denotes significant p-value (P < 0.05).

Parameters	Cases (n=60)	Controls (n=60)	P-value
	Mean±SD	Mean±SD	
IOP (mmHg)	20.56±7.72	14.28±2.31	<0.001*
ECD (cells/mm^2^)	2325.86±378.61	2527.083±197.05	<0.001*
AVG (μm^2^)	443.48±98.61	397.41±31.39	<0.001*
SD (μm^2^)	174±57.88	145.88±20.14	<0.001*
CV (%)	38.73±5.01	36.93±3.89	0.029*
MAX (μm^2^)	1111±325.66	958.4±300.54	0.008*
MIN (μm^2^)	112.7±41.26	98.1833±15.36	0.011*
HEX (%)	44.4±6.41	46.48±5.54	0.05
CCT (μm)	493.21±36.61	503.05±29.35	0.10

Subtype comparison: POAG versus PACG versus controls

PACG eyes exhibited a significantly lower ECD of 2177.52 ± 416.12 cells/mm² compared to POAG eyes (2405.74 ± 335.74 cells/mm²; P = 0.02) and controls (2527.08 ± 197.05 cells/mm²; P < 0.001). The AVG was markedly higher in PACG eyes (476.14 ± 117.77 μm²) than in POAG (425.89 ± 83.02 μm²; P = 0.05) and controls (397.41 ± 31.39 μm²; P < 0.001). SD was elevated in PACG (190.80 ± 67.11 μm²) compared to POAG (164.94 ± 50.90 μm²) and controls (145.88 ± 20.14 μm²), though the PACG vs POAG difference was not statistically significant (P = 0.09). CV was highest in PACG (39.61 ± 5.28%), followed by POAG (38.25 ± 4.85%) and controls (36.93 ± 3.89%), with PACG vs POAG comparison showing no significance (P = 0.31). MAX was greater in PACG (1197.28 ± 322.43 μm²) than POAG (1064.53 ± 321.90 μm²) and controls (958.40 ± 300.54 μm²), while MIN was also slightly higher in PACG (113.42 ± 32.72 μm²) than POAG (112.30 ± 45.60 μm²) and controls (98.18 ± 15.36 μm²); however, PACG vs POAG differences for MAX and MIN were not statistically significant (P = 0.13 and P = 0.92, respectively). HEX was lowest in PACG (42.42 ± 6.70%) and highest in controls (46.48 ± 5.54%), with a significant difference between PACG and controls (P = 0.007); the PACG vs POAG comparison approached significance (P = 0.07). CCT was similar across groups (PACG: 490.52 ± 23.21 μm; POAG: 494.66 ± 42.32 μm; controls: 503.05 ± 29.35 μm; P > 0.05) (Table 2).

**Table 2:** Comparison of IOP and specular microscopy parameters among POAG, PACG, and controls AVG, average cell area; CCT, central corneal thickness; CV, coefficient of variation; ECD, endothelial cell density; HEX, percentage of hexagonal cells; IOP, intraocular pressure; MAX, maximum cell area; MIN, minimum cell area; PACG, primary angle closure glaucoma; POAG, primary open angle glaucoma; SD, standard deviation *Denotes significant p-value (P < 0.05).

Parameter	PACG (n = 21 eyes)	POAG (n = 39 eyes)	Controls (n = 60 eyes)	PACG vs Controls (P-value)	POAG vs Controls (P-value)	PACG vs POAG (P-value)
IOP (mmHg)	23.52±10.17	18.97±5.54	14.28±2.31	<0.001*	<0.001*	0.028*
ECD (cells/mm²)	2177.52±416.12	2405.74±335.74	2527.08±197.05	<0.001*	0.025*	0.02*
AVG (μm²)	476.14±117.77	425.89±83.02	397.41±31.39	<0.001*	0.017*	0.05
SD (μm²)	190.80±67.11	164.94±50.90	145.88±20.14	<0.001*	0.010*	0.09
CV (%)	39.61±5.28	38.25±4.85	36.93±3.89	0.01*	0.138	0.31
MAX (μm²)	1197.28±322.43	1064.53±321.90	958.40±300.54	0.002*	0.098	0.13
MIN (μm²)	113.42±32.72	112.30±45.60	98.18±15.36	0.005*	0.028*	0.92
HEX (%)	42.42±6.70	45.46±6.07	46.48±5.54	0.007*	0.39	0.07
CCT (μm)	490.52±23.21	494.66±42.32	503.05±29.35	0.08	0.246	0.67

Severity-based comparison of glaucoma eyes

Glaucoma eyes were further stratified into early, moderate, and severe stages based on the Hodapp-Parrish-Anderson criteria. Perimetry was not feasible in nine eyes due to poor visual acuity. The prevalence of early, moderate, and severe glaucoma was 13 (25.49%), 12 (23.52%), and 26 (50.98%), respectively. Mean ECD declined with increasing severity (early: 2360.15 ± 340.90; moderate: 2339.33 ± 406.81; severe: 2258.69 ± 421.15 cells/mm²), though it was not significantly (P = 0.71). AVG increased across severity stages, but differences were not statistically significant (P = 0.93). SD, CV, MAX, and MIN were lowest in the early stage and highest in the severe stage, without significant differences (P > 0.05). HEX showed a decreasing trend (P = 0.20), and CCT remained comparable (P = 0.11) (Table 3).

**Table 3:** Comparison of specular microscopic parameters based on the severity of glaucoma AVG, average cell area; CCT, central corneal thickness; CV, coefficient of variation; ECD, endothelial cell density; HEX, percentage of hexagonal cells; MAX, maximum cell area; MIN, minimum cell area; SD, standard deviation Significant p-value (P < 0.05).

Parameters	Early(n=13)	Moderate(n=12)	Severe (n=26)	P-value
	Mean±SD	Mean±SD	Mean±SD	
ECD (cells/mm^2^)	2360.15±340.90	2339.33±406.81	2258.69±421.15	0.71
AVG (μm^2^)	435.23±88.78	441.5±121.13	459.34±106.45	0.93
SD (μm^2^)	163.38±47.12	173.16±67.88	187.76±64.98	0.48
CV (%)	37.30±4.17	38.91±4.62	40.19±5.76	0.26
MAX (μm^2^)	1033.61±255.27	1214.33±376.45	1155±359.73	0.39
MIN (μm^2^)	112±24.77	107.16±33.91	120.92±54.01	0.63
HEX (%)	47.07±6.62	43.5±5.10	43.03±7.36	0.20
CCT (μm)	506.46±34.17	495.91±36.80	481.30±35.80	0.11

Antiglaucoma medication use and duration

In cases, 24 (40%) were on three topical AGMs, indicating a significant reliance on multiple medications, 12 (20%) were on one AGM, and 14 (23.33%) were on two AGMs.

**Figure 1:**
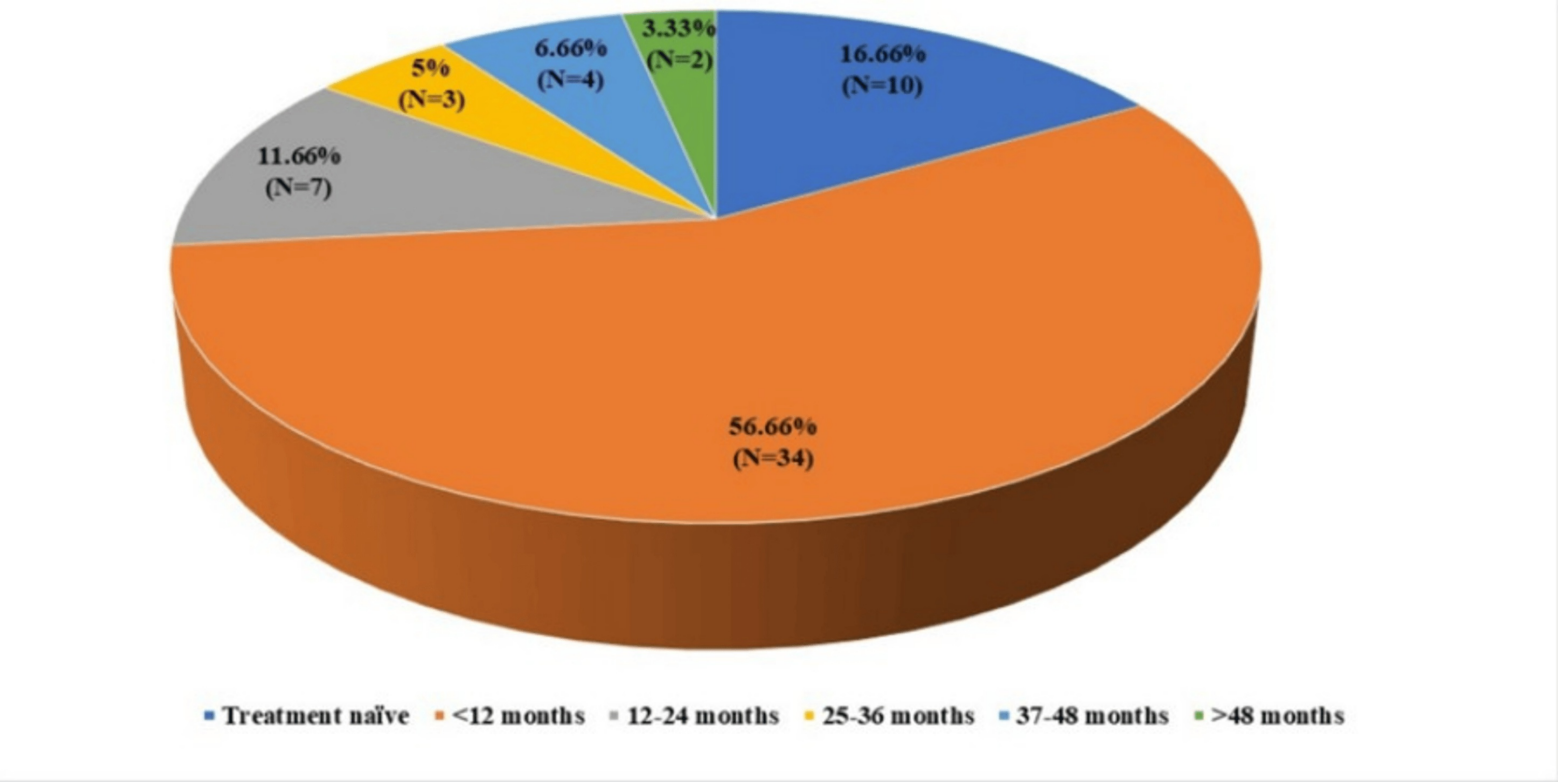
Distribution of cases based on duration of antiglaucoma medication use N=number of cases

Among the cases, 10 (16.66%) were treatment-naïve, having not received any prior treatment. The majority of cases, 34 (56.66%), had a disease duration of less than 12 months. A smaller percentage of patients, seven (11.66%), had AGM for 12-24 months. The duration ranged from 25 to 36 months in three (5%) of the cases and from 37 to 48 months in four (6.66%) of the cases. Lastly, two (3.33%) cases had AGM for more than 48 months (Figure 1).

Corneal endothelial changes in treatment-naïve eyes

The corneal endothelial parameters were also compared between treatment-naïve primary glaucoma patients and the control group. The mean ECD was significantly lower (P < 0.001) in treatment-naïve patients (2105.8 ± 524.05 cells/mm²) compared to controls (2527.083 ± 197.05 cells/mm²). In contrast, the mean AVG was significantly elevated (P < 0.001) in treatment-naïve primary glaucoma patients (506.6 ± 143.73 μm²) compared to controls (397.41 ± 31.394 μm²). The mean SD exhibited larger variability in treatment-naïve patients (207.4 ± 90.02 μm²) compared to controls (145.88 ± 20.148 μm²) (P < 0.001). The mean MAX and MIN were considerably greater (P < 0.05) in treatment-naïve patients compared to controls. The mean CV, HEX, and CCT were comparable between groups (Table 4).

**Table 4:** Comparison of specular microscopic parameters between treatment-naïve patients and the control group AGM, antiglaucoma medication; AVG, average cell area; CCT, central corneal thickness; CV, coefficient of variation; ECD, endothelial cell density; HEX, percentage of hexagonal cells; MAX, maximum cell area; MIN, minimum cell area; SD, standard deviation *Denotes significant p-value (P < 0.05).

Parameters	Treatment-naïve patients (n=10)	Controls (n=60)	P-value
	Mean±SD	Mean±SD	
ECD (cells/mm^2^	2105.8±524.05	2527.083±197.05	<0.001*
AVG (μm^2^)	506.6±143.73	397.41±31.39	<0.001*
SD (μm^2^)	207.4±90.02	145.88±20.14	<0.001*
CV (%)	39.6±7.08	36.93±3.89	0.08
MAX (μm^2^)	1263.4±397.36	958.4±300.54	0.006*
MIN (μm^2^)	138.7±44.99	98.1833±15.36	<0.001*
HEX (%)	43.2±8.79	46.48±5.54	0.11
CCT (μm)	496.7±40.21	503.05±29.35	0.55

Corneal endothelial changes based on AGM burden

Corneal endothelial parameters were compared among different groups based on the number of AGMs in patients with primary glaucoma. The mean ECD showed a declining trend, with the highest density recorded in the one AGM group at 2507.5 ± 189.89 cells/mm², followed by 2460.85 ± 267.02 cells/mm² in the two AGM group, and the lowest density in the three-AGM group at 2248 ± 383.44 cells/mm², with a statistically significant difference (P = 0.038). The mean AVG and mean SD increased with an increase in the number of AGMs, ranging from 398.5 ± 30.12 to 458.5 ± 106.62 μm² (P = 0.070) and 148.66 ± 16.43 to 183.54 ± 59.70 μm² (P = 0.061), respectively. The mean CV remained relatively stable across groups (37.33 ± 2.49% to 39.70 ± 4.81%, P = 0.233). The mean MAX and MIN showed no significant differences (P = 0.894 and 0.208, respectively). The mean HEX decreased with an increase in the number of AGMs (47 ± 4.97% to 42.79 ± 5.69%, P = 0.089). CCT remained consistent across groups (P = 0.347) (Table 5).

**Table 5:** Comparison of specular microscopic parameters between groups based on the number of antiglaucoma medications AGM, antiglaucoma medication; AVG, average cell area; CCT, central corneal thickness; CV, coefficient of variation; ECD, endothelial cell density; HEX, percentage of hexagonal cells; MAX, maximum cell area; MIN, minimum cell area; SD, standard deviation *Denotes significant p-value (P < 0.05)

Parameters	Patient on 1 AGM (n=12)	Patient on 2 AGMs (n=14)	Patient on 3 AGMs (n=24)	P-value
	Mean±SD	Mean±SD	Mean±SD	
ECD (cells/mm^2^)	2507.5±189.89	2460.85±267.02	2248±383.44	0.038*
AVG(μm^2^)	398.5±30.12	411.21±49.84	458.5±106.62	0.070
SD(μm^2^)	148.66±16.43	155.5±32.78	183.54±59.70	0.061
CV (%)	37.33±2.49	37.64±5.24	39.70±4.81	0.233
MAX(μm^2^)	1045.08±245.54	1082.85±345.21	1096.87±317.84	0.894
MIN(μm^2^)	95±15.39	101.21±18	117.41±52.14	0.208
HEX (%)	47±4.97	45.78±6.39	42.79±5.69	0.089
CCT(μm)	495.25±25.45	502.71±40.71	485.20±37.80	0.347

## Discussion

This study demonstrates that primary glaucoma is associated with significant corneal endothelial alterations, including reduced ECD, increased AVG, and greater variability in cell morphology. These findings reinforce prior evidence that glaucoma affects not only the optic nerve but also the corneal endothelium [5,11,12]. The absence of a significant difference in CCT between cases and controls suggests that subclinical endothelial dysfunction may precede overt corneal edema, highlighting the limitations of CCT as a sole indicator of endothelial health.

Endothelial loss was more pronounced in PACG compared to POAG, consistent with earlier studies [13,14]. This disparity likely reflects multifactorial mechanisms, including episodic angle closure, sustained IOP elevations, chronic iridocorneal contact, and impaired aqueous outflow, all of which may compromise endothelial oxygenation and nutrient delivery [14,15]. Although laser peripheral iridotomy (LPI) has been implicated in endothelial damage [16], longitudinal studies suggest that age-related decline is a more significant contributor to ECD loss than LPI itself [17].

Interestingly, treatment-naïve glaucoma patients exhibited significantly lower ECD compared to controls, indicating that glaucoma itself may contribute to endothelial damage independent of topical therapy. Chronic IOP elevation likely imposes metabolic stress on endothelial cells. While transient pressure spikes may be tolerated, sustained elevation can lead to irreversible cell loss [18]. These findings underscore the importance of early diagnosis and timely intervention to preserve endothelial integrity.

Medication burden appeared to further exacerbate endothelial compromise. Patients receiving multiple topical AGMs demonstrated lower ECD and reduced HEX compared to those on fewer agents. Similar trends have been reported by Yu et al. [19], whereas Elagamy et al. [20] found no significant association, possibly due to differences in drug formulations or treatment duration. Preservatives, particularly benzalkonium chloride (BAK), are known to disrupt tight junctions and induce oxidative stress, contributing to dose-dependent endothelial toxicity [21]. Comparative studies suggest that preservative-free formulations are generally safer, implicating preservatives rather than active compounds as the primary drivers of medication-related damage [21]. It is likely that both disease-related factors and chronic topical therapy act synergistically to accelerate endothelial decline.

Clinically, these findings highlight the importance of routine endothelial monitoring in glaucoma patients, particularly those with PACG or on long-term multi-drug regimens. Regular specular microscopy can aid in early detection of endothelial compromise and inform therapeutic decisions. Where feasible, preservative-free formulations should be preferred to minimize toxicity. Furthermore, endothelial assessment should be incorporated into preoperative planning for cataract or glaucoma surgery, as compromised corneas carry a higher risk of postoperative decompensation, especially in procedures where endothelial reserve is critical [14,20].

This study is not without limitations. Its cross-sectional nature precludes the establishment of causal relationships, and the relatively small sample size limits the generalizability of the findings. Additionally, the duration and formulation of topical medications (preserved vs preservative-free) were not analyzed, which may have introduced potential confounding factors. As the study population was hospital-based, selection bias cannot be fully excluded.

Future longitudinal studies with larger sample sizes are needed to better define the dynamic relationship between glaucoma, its treatment, and endothelial health. In particular, direct comparisons between preserved and preservative-free AGMs will help clarify whether endothelial toxicity is primarily attributable to preservatives or the pharmacologic agents themselves. Such insights will be crucial for optimizing long-term treatment strategies while minimizing corneal risk.

## Conclusions

Primary glaucoma is associated with significant corneal endothelial compromise, with PACG showing greater vulnerability than POAG. Both disease-related factors, such as sustained IOP elevation, and treatment-related factors, including cumulative exposure to topical AGMs, may contribute to endothelial loss. These findings emphasize the importance of incorporating routine endothelial assessment in glaucoma management, particularly in patients on long-term or multi-drug therapy. However, the cross-sectional design precludes confirmation of causal relationships, and future longitudinal studies are needed to validate these associations and clarify the effects of disease progression and treatment on endothelial health.
